# Foxp3^+^ Regulatory and Conventional CD4^+^ T Cells Display Similarly High Frequencies of Alloantigen-Reactive Cells

**DOI:** 10.3389/fimmu.2019.00521

**Published:** 2019-03-19

**Authors:** Mélanie Lalfer, Pascal Chappert, Maxime Carpentier, Dominique Urbain, Jean M. Davoust, David-Alexandre Gross

**Affiliations:** ^1^Institut National de la Santé et de la Recherche Médicale U1151 - Centre National de la Recherche Scientifique UMR 8253, Institut Necker-Enfants Malades, Paris, France; ^2^Université Paris Descartes, Sorbonne Paris Cité, Paris, France

**Keywords:** T cells, Foxp3^+^ regulatory T cells, TCR, alloreactivity, transplantation

## Abstract

Foxp3^+^ regulatory T cells (Tregs) play a major role in acquired immune tolerance to allogenic transplants. Their suppressive activity is thought to require T cell receptor (TCR)-driven antigen recognition; little, however, is known about the fraction of Tregs able to recognize alloantigens within this T cell subset primarily educated against self-antigens. Performing transfer experiments of Tregs or conventional T cells (Tconv) into both lymphoreplete and lymphopenic mice, we observed a similarly high proportion of cells signaling through their TCR and proliferating in allogenic hosts. Furthermore, using an *in vivo* proliferation assay with limited T cell numbers infused into lymphopenic mice, we found that the overall frequency of alloreactive Tregs was similar if not higher to that of alloreactive Tconv. Overall our study highlights a noticeably high level of alloreactive Foxp3^+^ regulatory T cells accounting for their predominant role in transplantation tolerance.

## Introduction

Foxp3^+^ regulatory T cells (Tregs) play a crucial role in the control of autoreactive T cells and in the maintenance of the immune system homeostasis (1–3). Beyond this critical role as regulators of self-tolerance, Tregs were also shown in mice to prevent or delay allograft rejection (4, 5) and suppress graft vs. host disease (GvHD) after allogenic bone marrow transplantation (6–9). The importance of regulatory T cells, in particular CD4^+^CD25^+^ T cells, exerting dominant tolerance against allograft was discovered more than three decades ago (10–12) and it was proposed early on to use these cells as a means to establish tissue-specific tolerance obviating or minimizing the need for immunosuppressive regimens (13). Adoptive immunotherapy with Tregs is now amenable in human for allogeneic hematopoietic stem cell transplantation, with recent clinical results reporting the prevention of acute and chronic GvHD (14) without loss of graft-vs. leukemia effect (15).

Optimal Tregs suppressive function relies on antigen (Ag) recognition as shown by their continuous T cell receptor (TCR) signaling requirement (16, 17) as well as by multiple evidences for their tissue specificity in models of autoimmunity (18), pregnancy (19), or cancer (20). The importance of Tregs specificity was also demonstrated in transplantation, as Tregs expanded with allogenic presenting cells proved to be more efficient than Tregs expanded with autologous cells or anti-CD3 beads to control GvHD (21) and prevent allograft rejection (5, 9).

The large fraction of alloreactive cells present within conventional CD4^+^ T cells (Tconv) has been appreciated for a long time as being responsible for organ rejection (22, 23) and is estimated to range between 1 and 10% of the entire T cell repertoire (24, 25). Little is known, however, about the proportion of alloreactive T cells within the Tregs compartment, which has a highly diversified TCR repertoire (26). Given the importance of their TCR in maintaining tolerance to self-antigens at the steady state and given the major role of Tregs in transplantation tolerance, we hypothesized that the repertoire of Tregs is also endowed with a substantial level of alloreactivity. In this study, we compared Tregs and Tconv side by side, and found that a similar proportion of both populations are able to signal through their TCR and expand in allogenic recipients.

## Materials and Methods

### Mice

All mice were used between 8 and 20 weeks of age. MHC haplotype antigens of C57Bl/6 (B6) mice are H-2K^b^, H-2D^b^, I-A^b^ (H-2^b^). MHC haplotype of NOD and NSG mice are H-2K^d^, H-2D^b^, I-A^g7^ (H-2^g7^). B6-Foxp3^EGFP^ (*Foxp3*^*tm*1*Mal*^) and NOD-Foxp3^EGFP^ (*Foxp3*^*tm*1*Kuch*^) are normal mice with *IRES-eGFP* knock-in into the Foxp3 locus on the B6 and NOD backgrounds, respectively. B6-RAG (*rag2*^−/−^) and NOD-RAG (*rag2*^−/−^) mice are defective in T and B cells. B6-RAGγc (*rag2*^−/−^ *IL2Rg*^−/−^) and NSG (*prkdc*^*scid*^ *IL2Rg*^−/−^) are defective in T, B and NK cells. B6xNOD F1 mice were generated by crossing B6 mice with NOD mice. All mice were housed under specific pathogen-free conditions and handled in accordance with French and European directives. Animal experiments were approved by the Ethical Committee of Paris Descartes University (CEEA 34).

### Cell Isolation

CD4^+^ T cells were enriched from pooled splenocytes and inguinal, brachial, and cervical lymph node cells using Dynal CD4 Negative Isolation Kit (Dynal Biotech). Viable CD4^+^ Foxp3-EGFP^+^ (Tregs) and CD4^+^ Foxp3-EGFP^−^ (Tconv) cells were further sorted on a FACSAria (Becton Dickinson) or Sony SH800 (Sony). A purity of >99.9% Tregs or Tconv was regularly achieved. Of note, TCR Vβ3^+^ cells were excluded from the cell-sorted B6 CD4^+^ population to avoid potentially confounding superantigen-related stimulation systematically encountered in NSG hosts (data not shown). Cells were injected into the retro-orbital venous sinus in 0.2 ml PBS 1X.

### FACS Analysis

Surface staining were performed in PBS 1X, with 2 mM EDTA and 0.1% BSA. Cell suspensions were first incubated with anti-FcγRIII/II (2.4G2, BioXcell) mAb for 15 min at 4°C and then stained for 30 min at 4°C with saturating amounts of indicated combinations of PE or AF700 anti-CD45.2 (104), e450, APC or PE-Cy7 anti-CD45.1 (A20), FITC, APC or PE-Cy7 anti-TCRβ (H57-597), e450, V500, or PE anti-CD4 (RM4-5). Dead cells were excluded using 7-actinomycin D (Sigma-Aldrich). For Nur77 and Foxp3 co-staining, cells were fixed 30 min at 4°C using Foxp3 Fixation/Permeabilization buffer (eBioscience) and labeled overnight with PE-conjugated anti-Nur77 (12–14) and APC-conjugated anti-Foxp3 mAb (FJK-16s) in Permeabilization Buffer (eBioscience). Antibodies were purchased from Becton Dickinson, eBioscience, or Sony Biotechnology. Dead cells were excluded using LIVE/DEAD® Fixable Near-IR staining (Life Technologies). VPD450-labeling (1 μM) was performed according to manufacturer instructions (Becton Dickinson).

### Statistics

For all statistical analyses, an unpaired *t*-test was performed within Excel software. In **Figures 3A,C**, **4C**, mice were considered as negative (and plotted on the x axis) when the percentage of positive events found by FACS analysis in experimental group was inferior to three times the percentage of positive events detected in non-transferred NSG mice. This led to a detection limit of ~100 cells per spleen. Limiting dilution frequencies were calculated using L-Calc software (Stem-Cell Technologies).

## Results

### Activation and Expansion of Both Tconv and Tregs in Allogenic Hosts

**Figure 1:**
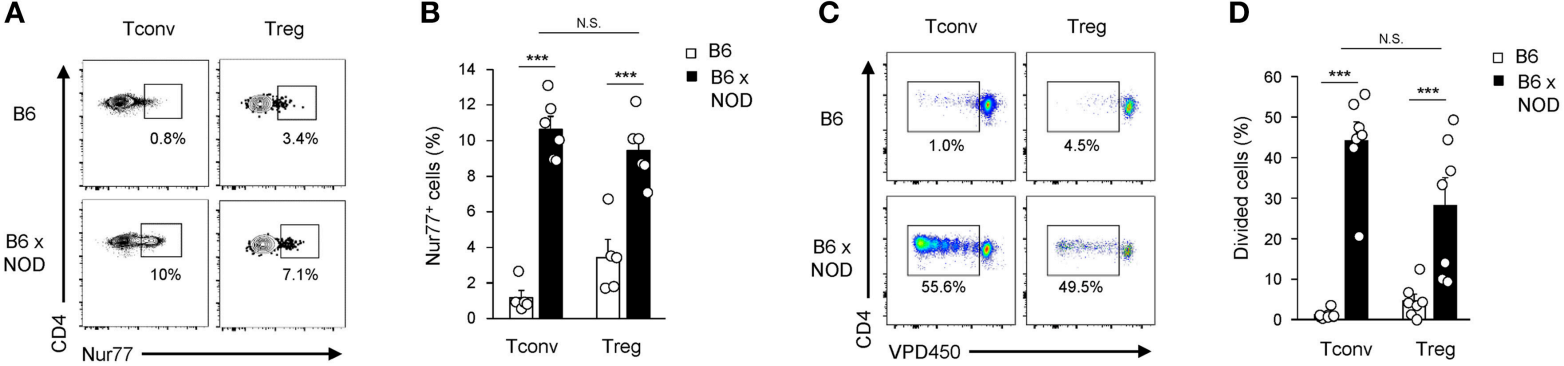
Activation and expansion of Tconv and Tregs in allogenic lymphoreplete hosts. **(A,B)** 20–40 × 10^6^ B6 splenocytes (CD45.2) were transferred into syngenic B6 CD45.1 vs. allogenic B6xNOD (CD45.2/CD45.1) mice. Five hours post-transfer, mice were sacrificed and their splenocytes analyzed by FACS using AF700 anti-CD45.2, PE-Cy7 anti-CD45.1, FITC anti-TCRβ, e450 anti-CD4, PE anti-Nur77, and APC anti-Foxp3 mAb. **(A)** Representative dot plots gated on either Foxp3^−^ (Tconv) or Foxp3^+^ (Tregs) live donor CD45.1^−^CD45.2^+^TCRβ^+^CD4^+^ cells and **(B)** percentages of Nur77^+^ cells in donor T cells recovered from indicated host are shown. **(C,D)** 20–30 × 10^6^ VPD450-labeled Foxp3-EGFP splenocytes (CD45.2) were transferred into B6 CD45.1 vs. B6xNOD mice. Three days post-transfer, mice were sacrificed and their splenocytes analyzed by FACS using PE anti-CD45.2, APC anti-CD45.1, PE-Cy7 anti-TCRβ and V500 anti-CD4 mAb. **(C)** Representative dot plots gated on either Foxp3EGFP^−^ or Foxp3EGFP^+^ live donor CD45.1^−^CD45.2^+^TCRβ^+^CD4^+^ cells and **(D)** percentages of divided donor T cells in individual mice are shown. Data are pooled from 3 to 4 independent experiments and represented as mean ± standard error of the mean (SEM). NS, non-significant; ***p < 0.001.

Alloreactivity of Tconv and Tregs cells was assessed using adoptive transfer of T cells from C57Bl/6 (B6) mice (H-2^b^) into MHC-mismatched NOD mice (H-2^g7^). We first choose to measure both the upregulation of Nur77, an immediate early gene up-regulated by TCR stimulation in thymocytes and T cells shown to correlate nicely with TCR signal strength (27), and subsequent cell proliferation events. Splenocytes from B6 CD45.2^+^ mice were transferred into congenic B6 CD45.1^+^ mice or allogenic B6xNOD CD45.1^+/−^ (B6xNOD) mice. These latter F1 mice were used as recipients to prevent NK cell-mediated rejection of donor T cells. As expected, Nur77 was induced in a higher proportion of Tconv transferred into allogenic hosts (10.6 ± 0.7%) compared to syngenic hosts (1.2 ± 0.4%) (Figures 1A,B). Importantly, a similar proportion of Tregs also upregulated Nur77 upon transfer into allogenic recipients (9.5 ± 0.8%), while a basal level of Nur77 upregulation was detected following transfer into syngenic mice (3.4 ± 1%), probably due to self-reactivity (27). In line with the comparable rates of early TCR signaling events detected in both populations, similar proliferation profiles were detected in both Tconv and Tregs 3 days after transfer into allogenic hosts (Figures 1C,D). Altogether, these results indicate that a similar, and sizeable, proportion of Tregs and Tconv, experience TCR-driven alloantigen recognition upon transfer in lymphoreplete hosts.

**Figure 2:**
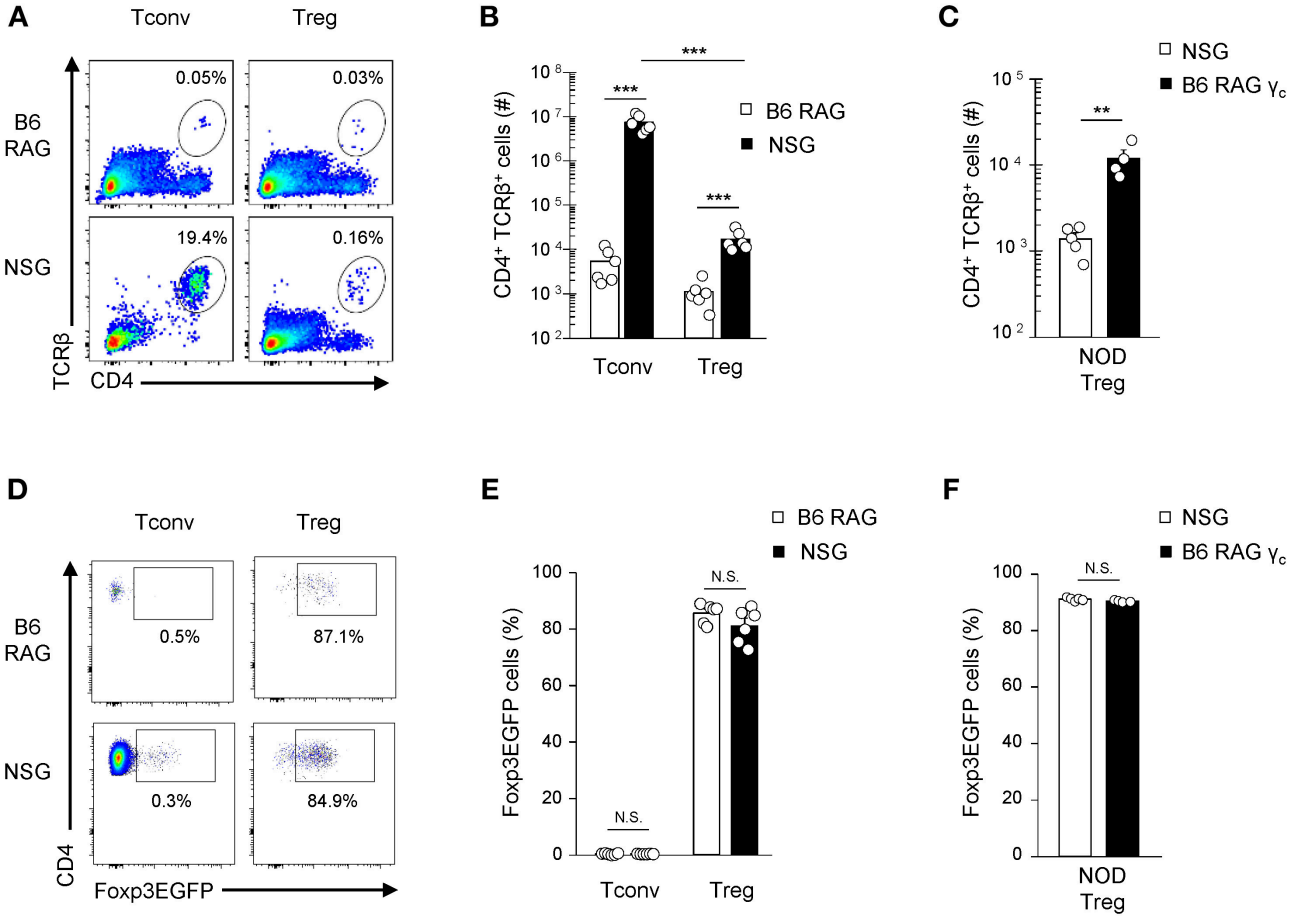
Expansion of both Tconv and Tregs in allogenic lymphopenic hosts. 1 × 10^5^ B6-Tconv or Tregs were cell-sorted from B6-Foxp3^EGFP^ mice and transferred into B6-RAG vs. NSG mice **(A,B,D,E)**. 1 × 10^5^ NOD-Tregs were cell-sorted from NOD-Foxp3^EGFP^ mice and transferred into NSG vs. B6-RAGγc mice **(C,F)**. Mice were sacrificed at day 7 and their splenocytes analyzed by FACS using PE anti-CD45.2, APC anti-CD45.1, PE-Cy7 anti-TCRβ and V500 anti-CD4 mAb. **(A)** Representative dot plots gated on live cells and **(B,C)** absolute numbers of recovered CD4^+^TCRβ^+^Foxp3EGFP^−^ Treg and/or Foxp3EGFP^+^ Tconv in individual mice are shown. **(D)** Representative dot plots and **(E,F)** percentages of Foxp3EGFP expressing cells gated on CD4^+^TCRβ^+^ live cells. Data are pooled from 3 to 4 **(A,B,D,E)** or 1 **(C,F)** independent experiments and represented as mean ± standard error of the mean (SEM). NS, non-significant; **p < 0.01, ***p < 0.001.

Interestingly, transfer into lymphopenic hosts avoids potential competition with other resident T cells (28) and mimics the lymphopenia conditions associated with transplant regimen. We therefore explored the expansion of Foxp3-EGFP^hi^-sorted B6 Tregs transferred into either allogenic NOD Prkdc^scid^ IL2R$\gamma_{\text{c}}^{-/-}$ (NSG) or syngenic C57Bl/6 Rag2^−/−^ (B6-RAG) mice. IL2R$\gamma_{\text{c}}^{-/-}$ mutation present in NSG mice was instrumental to prevent NK cell development and NK cell-mediated rejection of allogenic donor T cells. One week after transfer, twenty fold more Tregs were recovered in NSG mice as compared to B6-RAG mice (Figures 2A,B). As previously observed in lymphoreplete hosts, this was correlated with higher Nur77 expression and extensive proliferation up to day 7 for both Tregs and Tconv (data not shown). Reciprocal transfer experiments (H-2^g7^ -> H-2^b^) were performed to ensure that this alloreactive potential of Tregs is not specific to B6 Tregs and does not depend on particular hosts combinations. Confirming our previous findings, higher expansion of Tregs sorted from NOD-Foxp3^EGFP^ mice was observed after transfer into allogenic B6-RAGγc recipients compared to syngenic NSG (Figure 2C) or NOD-RAG recipients (data not shown). Of importance, Foxp3-EGFP expression was maintained in more than 80% of Tregs in both syngenic and allogenic recipients, attesting their *in vivo* stability (Figures 2D–F). As expected, Tconv cells underwent strong expansion in NSG mice (Figures 2A,B), and almost no conversion of Tconv cells into Treg cells was observed in this setting (Figures 2D–E).

### Frequencies of Alloreactive Tconv and Tregs Are Similarly High

**Figure 3:**
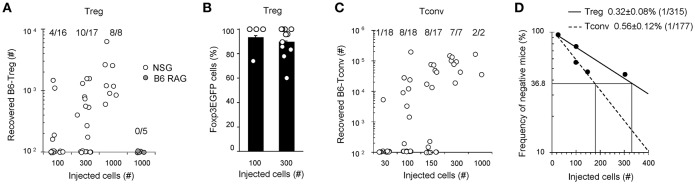
Frequencies of alloreactive Tconv and Tregs. Indicated numbers (30–1000) of cell-sorted Tregs or Tconv were transferred into NSG mice. At day 7, mice were sacrificed and their splenocytes analyzed by FACS using APC anti-TCRβ and PE anti-CD4 mAb. **(A,C)** Absolute numbers of recovered live CD4^+^TCRβ^+^Foxp3^+^ Tregs and CD4^+^TCRβ^+^Foxp3^−^ Tconv in individual mice are shown. **(B)** Percentage of Foxp3EGFP^+^ cells into individual mice injected with 100 or 300 Tregs that exhibit CD4^+^TCRβ^+^ events. **(D)** Numbers of injected T cells are plotted against the log frequency of negative mice. Mean ± standard error of the mean (SEM) is shown. Data are pooled from 4 independent experiments.

To gain access to the frequency of alloreactive Tregs, we next performed an *in vivo* limiting dilution assay (LDA) in lymphopenic hosts, using low numbers of B6 T cells transferred into NSG or control B6-RAG mice. Seven days post-transfer, Tregs were found in a fraction of NSG mice receiving as little as 100 Tregs (4 out of 16 mice), while none were found in B6-RAG hosts receiving 10-times more Tregs, confirming at low cell numbers the predominant role of allogenic stimulation for Tregs expansion (Figure 3A). Indeed, the proportion of NSG mice in which Tregs were detected increased when more cells were injected. Linear regression of the frequency of negative mice vs. the numbers of injected T cells gave a proportion of 0.32 ± 0.08% of injected Tregs (*f* = 1/315) expanding in allogenic recipients (Figure 3D). Interestingly, in line with the high percentage of Foxp3^+^ cells observed upon transfer of high cell numbers (Figures 2D–F), Foxp3 expression was equally maintained at low numbers of injected Tregs (Figure 3B). This experiment was repeated with Tconv and a closely related percentage of 0.56 ± 0.12% alloreactive Tconv (*f* = 1/177) was found (Figures 3C,D).

**Figure 4:**
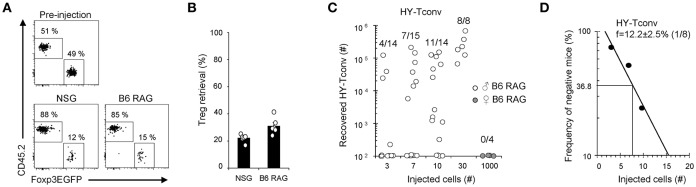
Adoptive transfer survival of Tconv and Tregs in lymphopenic hosts. **(A,B)** 5 × 10^4^ Tregs (CD45.1) were mixed with 5 × 10^4^ Tconv (CD45.2) and transferred into male NSG or B6-RAG mice. One day later, mice were sacrificed and their splenocytes analyzed by FACS using PE anti-CD4, APC anti-TCRβ, e450 anti-CD45.1 and AF700 anti-CD45.2 mAb. **(A)** Representative dot plots gated on live donor CD4^+^ TCRβ^+^ cells. **(B)** Histogram shows Tregs retrieval as a percentage of Tconv. Mean ± standard error of the mean (SEM) is shown. **(C,D)** Indicated numbers (3–1000) of CD4^+^ RAG2^−/−^ Marilyn T cells were transferred into male or female B6-RAG mice. At day 7, mice were sacrificed and their splenocytes analyzed by FACS using APC anti-TCRβ and PE anti-CD4 mAb. **(C)** Absolute numbers of recovered live CD4^+^TCRβ^+^ Marylin T cells in individual mice are shown. **(D)** Numbers of injected Marilyn T cells are plotted against the log frequency of negative mice. Data are pooled from 2 **(A,B)** or 4 **(C,D)** independent experiments.

Next, to compare Tregs and Tconv survival upon transfer into lymphopenic mice, 5 × 10^4^ Tconv and 5 × 10^4^ Tregs were co-injected into NSG or B6-RAG mice and their relative proportions determined 16 h later, prior to any cell expansion. At this early time point, Tregs recovery in secondary lymphoid organs amounted to 12.4 ± 0.8% and 18.4 ± 1.9% relative to that of Tconv in NSG and B6-RAG mice, respectively (Figures 4A,B). This lower survival was not due to cell-sorting procedure since direct injection of B6 splenocytes into NSG or B6-RAG mice yielded a comparable Tregs over Tconv survival ratio of 18.8 ± 2.3% and 19.5 ± 1.8%, respectively (data not shown). Of note, it has been reported that lower Tregs survival could result from ARTC2-mediated ADP-ribosylation of P2X7 during murine primary lymphocytes preparation (29). Finally, to estimate the absolute level of Tconv survival after transfer into lymphopenic hosts, we transferred limited amounts of Rag^−/−^ TCR transgenic Marilyn T cells, all reactive to the HY male antigen, and found that a proportion of 12.2 ± 2.5% (*f* = 1/8) of injected Marilyn Tconv survived and underwent expansion in male B6-RAG mice (Figures 4C,D).

Combining the survival ratio of Tregs over Tconv (Figures 4A,B) with the survival of Marilyn Tconv (Figures 4C,D), our results indicate that only 1.5 ± 0.4% of Tregs (i.e., 12.4 ± 0.8% × 12.2 ± 2.5%) were able to survive after adoptive transfer into NSG mice. This survival efficiency is markedly different from that of Tconv, leading to corrected alloreactive frequencies for Tregs and Tconv of 21.0 ± 10.7% (0.32 ± 0.08% over 1.5 ± 0.4% surviving cells) and 4.6 ± 1.9% (0.56 ± 0.12% over 12.2 ± 2.5% surviving cells), respectively.

## Discussion

To compare the frequency of alloreactive Tregs and Tconv *in vivo*, we used two different methods: an indirect measure of TCR activation (Nur77) and a limiting dilution assay. While powerful, LDA has rarely been used *in vivo*, because of two experimental constraints. First, detection of limited number of T cells is laborious in normal hosts. Second, classical analysis at 4 weeks takes into account proliferation events resulting from both cognate MHC/antigen-TCR interactions and the so-called homeostatic proliferation, which is mainly driven by available environmental resources (30).

Using lymphopenic hosts and focusing on early time points, we estimated that 4.6 ± 1.9% of Tconv that survived the initial transfer underwent alloantigen-driven expansion. This result is in the same range, and somewhat lower, than the frequency found using Nur77 measurement in lymphoreplete hosts (10.6 ± 0.7%), and is in agreement with other studies using different approaches such as measurement of prolonged interactions using time-lapse microscopy (5–15%) (31), CFSE dye dilution after *in vivo* transfer (0.7–21% depending on the calculation method) or *in vitro* mixed lymphocyte reactions (4.0–4.6%) (24, 25).

Regarding Treg cells, a similarly high frequency of alloreactive cells was detected *in vivo* by Nur77 measurement (9.5 ± 0.8%), in agreement with a previous study using an *in vitro* analysis of the reactivity of hybridoma cells derived from a limited set of Tconv and Tregs (32). Taking into account the lesser survival efficiency of Tregs, the percentage of alloreactive Tregs (21.0 ± 10.7%) appears higher than that of Tconv (4.6 ± 1.9%). This difference largely depends on the survival measurement that could be affected by different parameters such as differential migration patterns into non-lymphoid tissues between Tregs and Tconv (33). Nevertheless, a higher alloreactivity was also suggested using an *in vivo* MLR, which does not take into account inhibitory effects due to T cell competition (34). Interestingly, no major difference were observed when other T cell subsets were compared: frequencies of alloreactive CD4^+^ and CD8^+^ were in the same range (3.9% for the CD4^+^, 2.5% for the CD8^+^), and naïve and memory cells contribute equally to the allo-response (35). Overall, assays based on early TCR signaling measurement (Nur77, hybridoma) give rise to underestimated fractions of alloreactive Tregs as compared to assays based on T cell expansion (LDA, MLR), a fact that could be explained by the particular TCR machinery of Tregs (36).

Our findings are mainly based on transfer of B6 Tregs into MHC-mismatched NOD mice (H-2^b^ -> H-2^g7^). Nevertheless, alloreactivity of Tregs has been noticed in other MHC combinations, with B6 Tregs alloreactivity being observed against H-2^d^ and H-2^k^ haplotypes (32, 34), and in our reciprocal transfer experiments (H-2^g7^ -> H-2^b^), which evidenced equally high alloreactive Tregs expansion (Figure 2C). Finally, it should be noted that to prevent NK cell-mediated rejection of allogenic T cells, we used IL2R$\gamma_{\text{c}}^{-/-}$ lymphopenic hosts, devoid of NK cells. Resulting from the absence of functional receptors, these mice could have increased levels of cytokines such as IL-7, which is important for Tregs maintenance (37). Here, significant differences were still observed for the expansion of NOD-Foxp3^EGFP^ Tregs adoptively transferred into allogenic B6-RAGγc (IL2R$\gamma_{\text{c}}^{-/-}$) mice compared to syngenic mice being either IL2R$\gamma_{\text{c}}^{-/-}$ (NSG) (Figure 2C) or IL2R$\gamma_{\text{c}}^{+/+}$ (NOD RAG) (data not shown), ruling out this potential non-MHC-dependent strain-specific effect.

The TCR repertoire of Tregs and Tconv are vast and diverse but poorly overlapping (26). Whether cross-reactivity that applies to the Tconv subset and explain their alloreactivity (38) equally applies to the Tregs compartment is unknown. The high frequency of allogenic Tregs found here can have different explanations. First, it has been shown that dual TCR T cells are predisposed to alloreactivity (39), and a vast majority of Tregs harbor two different TCRα chain (40). Second, Tregs are more resistant to deletional process in the thymus (41) and most of the TCRα chains that normally promote negative selection confer a high level of alloreactivity (42).

Our results also showed that frequency of Tregs reactive to self-Ags (B6 syngenic hosts) was lower than that reactive to alloantigens (NOD allogenic hosts), as measured by Nur77 expression, VPD450 dye dilution and early cell expansion capacity. Of note, Tregs emerge in the thymus upon agonist self-antigen recognition (43), require continuous TCR signaling to exert their function in the periphery (16, 17) and demonstrate tissue specificity in models of autoimmunity (18), pregnancy (19), or cancer (20), but these experimental observations provided only indirect proofs of recognition of self-Ags. Intriguingly, hybridoma cells derived from either Tregs (32) or “would be” Tregs (44) were found poorly reactive against syngenic splenocytes, and in contrast to Tconv, “would be” Tregs, which express a non-functional Foxp3 protein, were not pathogenic after transfer into lymphopenic mice (45, 46). However, two naturally-expressed Ags have been recently identified as being recognized by Tregs: TCAF3, a prostate-specific protein, which is expressed in the thymus in an Aire-dependant manner (47) and MOG, which is a minor component of the myelin expressed in the central nervous system (48). The fraction of Tregs recognizing such tissue-specific Ags is not known and it is possible that only a minor fraction of the Tregs pool possess this kind of antigenic specificity. Indeed, exogenous Ags-specific Tregs were also implicated in limiting pathological immunity toward environmental antigens including commensal bacteria (49) and allergens (50). Overall, two non-mutually exclusive parameters could explain the lower reactivity of Tregs toward self-Ags found here, even when assessed in recipient lymph nodes (data not shown): (1) lower and limiting abundance of self-peptides-MHC complexes as compared to allogenic MHC, (2) lower TCR avidity for self-Ags than for alloantigens.

Foxp3^+^CD4^+^ regulatory T cells, formerly known as CD4^+^CD25^+^, have been identified as a major component required for transplantation tolerance (51–53). These cells are mobilized by short-term immunosuppressive drugs and promote long-term tolerance through “linked suppression” and “infectious tolerance” mechanisms relying on their specific immunosuppressive properties (13). This advantageous Ag-specific activity of Tregs was also observed in other models, such as type 1 diabetes (54) or gene transfer (55), but depend on the expression of transgenic TCRs. In the transplantation field, protocols aiming at recruiting alloantigen-specific Tregs were particularly efficient to prevent both allograft rejection and GvHD in mice (5–9) and to promote tolerance induction in human (56, 57). Altogether, our results reveal an important level of alloreactivity within the vast self-specific Tregs repertoire, in line with and supporting their predominant role in controlling adverse immune responses in organ transplantation.

## Data Availability

All datasets generated for this study are included in the manuscript.

## Author Contributions

ML, PC, MC, and DU performed experiments. ML analyzed and prepared figures. PC, JD, and D-AG wrote the manuscript.

### Conflict of Interest Statement

The authors declare that the research was conducted in the absence of any commercial or financial relationships that could be construed as a potential conflict of interest.
